# Size effects of lamellar twins on the strength and deformation mechanisms of nanocrystalline hcp cobalt

**DOI:** 10.1038/s41598-017-09919-2

**Published:** 2017-08-25

**Authors:** Wen Wang, Fuping Yuan, Ping Jiang, Xiaolei Wu

**Affiliations:** 1 State Key Laboratory of Nonlinear Mechanics, Institute of Mechanics, Chinese Academy of Sciences, Beijing, 100190 China; 2 School of Engineering Science, University of Chinese Academy of Sciences, Beijing, 100049 People’s Republic of China

## Abstract

Twins play an important role in the deformation of nanocrystalline (NC) metals. The size effects of {$10\bar{1}2$} tensile/{$10\bar{1}1$} compressive lamellar twins on the tensile strength and deformation mechanisms of NC hcp cobalt have been investigated by a series of large-scale molecular dynamics simulations. Unlike the size effects of twins on the strength for polycrystalline fcc metals, the strength of NC hcp cobalt with lamellar tensile/compressive twins monotonically increases with decreasing twin boundary spacing (TBS) and no softening stage is observed, which is due to the consistent deformation mechanisms no matter TBS is large or small. These consistent deformation mechanisms can be categorized into four types of strengthening mechanisms: (i) Partial basal dislocations nucleated from grain boundaries (GBs) or twin boundaries (TBs) intersecting with TBs/GBs; (ii) Phase transformation from hcp to fcc; (iii) <c + a> partial edge dislocations nucleated from TBs intersecting with basal partial dislocations; (iv) Growth of the newly formed secondary tensile twins inside the primary compressive/tensile twins. The observed multiple twinning in MD simulations has also been confirmed by TEM after tensile testing in NC cobalt processed by severe plastic deformation.

## Introduction

Stronger and tougher metals and alloys have been the pursuit of scientists for structural applications for centuries although strength and ductility are in general mutually exclusive^1–11^. For example, ultrafine-grained (UFG) and nanocrystalline (NC) metals usually have much higher yield strength than that of coarse-grain (CG) counterpart, while show reduced strain hardening and limited uniform tensile elongation^2–6^. Recently, twinning at the nanoscale have been regarded as an efficient method to obtain both high strength and substantial ductility^12–14^.

Previous research have indicated deformation twins are more difficult to form with decreasing grain size for fcc metals in the CG size range^15^. However, twinning becomes easier with decreasing grain size when the grain size is below 100 nm although twinning might become difficult again once the grain size is too small (below 20 nm)^16–18^. In contrast, twinning is an important mechanism for plastic deformation of CG hcp metals in addition to dislocation slip due to their relatively limited slip systems when compared to fcc metals^19^. Theoretically, at least seven twinning modes involving different twinning planes can be existing in hcp metals, such as {$10\bar{1}2$}, {$10\bar{1}1$}, {$10\bar{1}3$}, {$11\bar{2}1$}, {$11\bar{2}2$}, {$11\bar{2}3$}, {$11\bar{2}4$} twins^20, 21^. Among them, {$10\bar{1}2$} and {$10\bar{1}1$} twins (usually referred to tensile twins and compressive twins) are the most common twinning modes in hcp metals and alloys^22, 23^, which can be easily observed in the $[11\bar{2}0]$ zone axis under TEM.

However, twinning becomes more difficult to form with decreasing grain size in hcp metals, especially deformation twins are rarely observed in NC hcp metals and alloys^19^. It is scientifically important to activate deformation twins in NC hcp metals since twinning can stimulate both high strength and good ductility^22–25^. Pure cobalt, with an hcp crystal structure at room temperature, has been experimentally observed to deform by twinning during the plastic deformation (especially at low temperatures and high strain rates)^26–31^, even in the nanoscale grain size range^30, 31^, due to its very low stacking faults energy (SFE, 27 ± 4 mJ/m^2^) compared to the other hcp pure metals, such as magnesium (50–80 mJ/m^2^), titanium (>300 mJ/m^2^) and zirconium (80 mJ/m^2^)^31^. Annealing twins have also been observed in pure cobalt during the electroplated process or the annealing process^24, 25^. Recently, multiple twinning has been found to play a key role in the plastic deformation and the grain refinement of hcp metals and alloys^21, 32^.

Molecular Dynamics (MD) simulations have proven to be powerful tools for studying the grain size effect on the strength and the atomistic deformation mechanisms of NC fcc^33, 34^, bcc^35^ and hcp^36–38^ metals, with carefully designed modeling cells in which the real-time responses of the microstructures can be examined. Experiments and MD simulations have also shown a transition in deformation mechanisms at the critical twin boundary spacing (TBS), i.e., from the classical Hall-Petch type strengthening due to the interaction between the dislocation and the twin boundary (TB) to a dislocation-nucleation-controlled softening mechanism with detwinning for fcc metals^14, 39^. It is technically important to investigate the TBS effect on the flow behaviors of NC hcp metals with nano-twins since twinning can stimulate excellent mechanical properties. However, the micro-structural deformation mechanisms of NC hcp metals with nano-twins still remain vague. In this regard, a series of large-scale MD simulations have been performed in the present study to investigate the size effect of twins on the strength and the related atomic-level deformation mechanisms of NC hcp cobalt, which will help to provide insights for achieving better mechanical properties in hcp metals and alloys with low SFE. Thus, $[11\bar{2}0]$-textured simulation cells with hexagonal columnar grains (Similar to the configuration used by Kim *et al*.^36^) were considered in the present study, as shown in Fig. S1. The typical configurations for nanocrystalline cobalt (*d* = 60 nm) with lamellar {$10\bar{1}2$} tensile twins (TBS = 8.32 nm) and with lamellar {$10\bar{1}1$} compressive twins (TBS = 8.45 nm) are shown in Fig. S1a,c, respectively. The corresponding close-up views for the rectangular areas in Fig. S1a,c showing the details for the lamellar {$10\bar{1}2$} tensile twins and the lamellar {$10\bar{1}1$} compressive twins are displayed in Fig. S1b,d, respectively. Six samples with TBS = 0.89, 2.38, 4.75, 8.32, 14.85, 23.76 nm for tensile twins and six samples with TBS = 0.77, 2.31, 4.61, 8.45, 15.37, 24.59 nm for compressive twins were simulated in order to investigate the TBS effect on the strength and the atomistic deformation mechanisms of NC cobalt. The same Voronoi grain structure and the same crystallographic orientations of all grains are retained as TBS changes. The other simulation details are described in Methods section.

## Results

### Stress-strain curves and TBS effect on the flow behaviors

**Figure 1:**
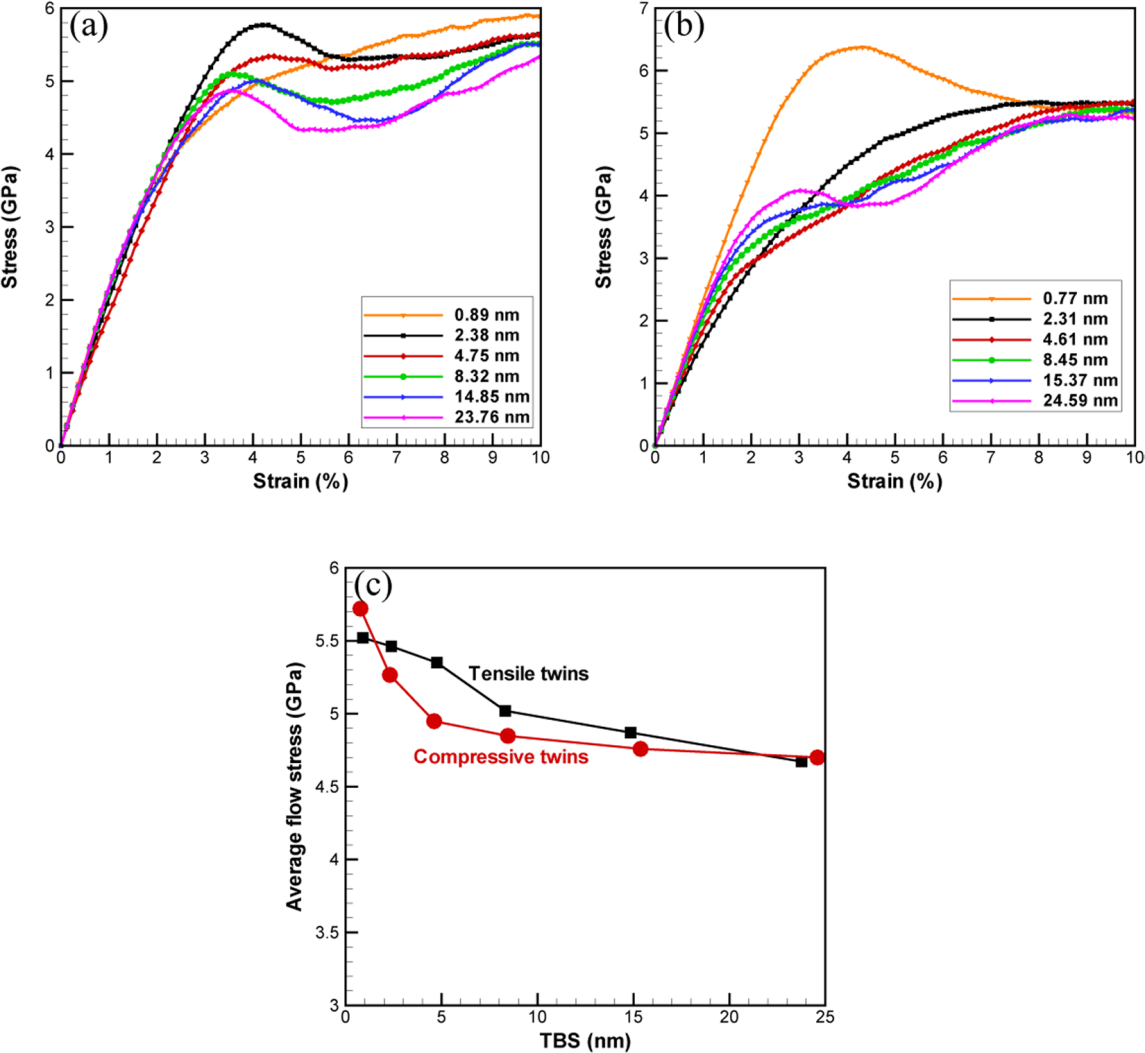
Simulated mechanical properties for NC hcp cobalt with lamellar twins. (**a**,**b**) Simulated stress-strain curves for NC hcp cobalt with lamellar {$10\bar{1}2$} tensile twins and lamellar {$10\bar{1}1$} compressive twins, respectively; (**c**) The average flow stress (strain between 4% and 10%) vs. TBS for NC hcp cobalt with lamellar twins.

In the NC hcp cobalt with nanotwins, there exist two characteristic microstructrual length scales (the grain size *d* and the TBS). In order to study the size effects of twins, the TBS is varied while the grain size is fixed at *d* = 60 nm in the present study. Figure 1a and b display the simulated stress-strain curves for various NC hcp Co samples with lamellar {$10\bar{1}2$} tensile twins or lamellar {$10\bar{1}1$} compressive twins, respectively. It should be noted that stress strain curves show different behaviors for NC hcp Co samples with lamellar {$10\bar{1}2$} tensile twins and lamellar {$10\bar{1}1$} compressive twins. In Fig. 1a, tensile stresses are observed to increase with strain linearly first to a peak value (onset of plastic deformation), then tensile stresses stay at a steady state with small fluctuations. While, in Fig. 1b, tensile stresses are also observed to increase linearly first in the elastic stage, then increase nonlinearly up to a steady-state value, showing strong strain hardening in the plastic stage before the final plateau. It is typically more meaningful to take the average value for the flow stress over a certain plastic strain interval^33–38^ in order to investigate the size effect of lamellar twins on the strength of NC hcp Co. In this regard, the average flow stress at strains between 4% and 10% is plotted against TBS in Fig. 1c. With the same grain size, it should be noted that the average flow stress monotonically increases with decreasing TBS for NC cobalt with both lamellar {$10\bar{1}2$} tensile twins and lamellar {$10\bar{1}1$} compressive twins, and there is no observed softening stage when the TBS is small. The TBS effect on the flow behavior of NC hcp cobalt is totally different from that of fcc metals^14, 39^, and the corresponding atomistic deformation mechanisms will be discussed in the following sections. As indicated in previous research^14^, the effects of both twin lamellar thickness and grain size on the strength of fcc metals are coupled, thus more studies should be conducted in the future work to illustrate the coupling effects of both twin lamellar thickness and grain size on the strength of NC hcp metals.

### Atomistic deformation mechanisms for NC hcp cobalt with tensile twins

**Figure 2:**
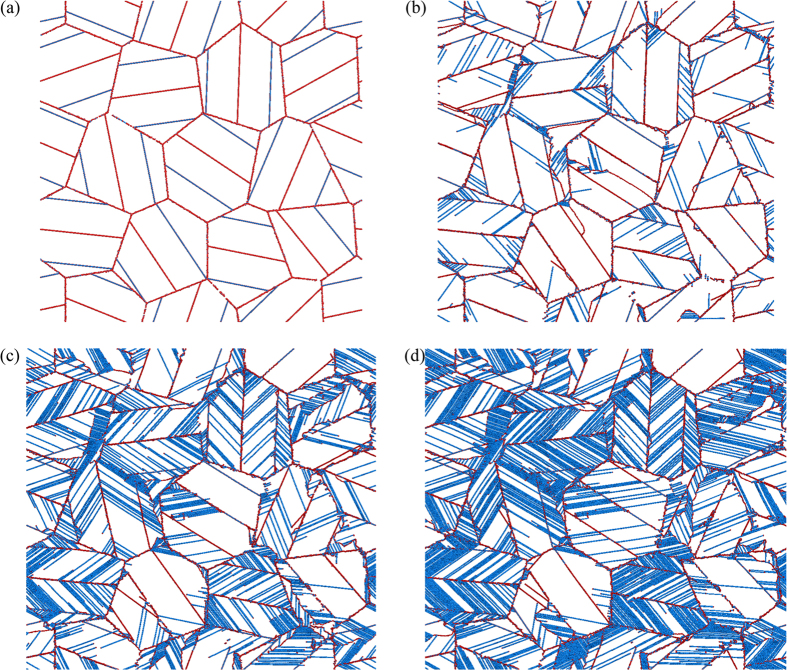
Simulated deformation patterns for NC hcp cobalt with lamellar {$10\bar{1}2$} tensile twins (TBS = 23.76 nm). The snapshots were taken at strains of (**a**) 0%; (**b**) 3%; (**c**) 5%; (**d**) 7%.

**Figure 3:**
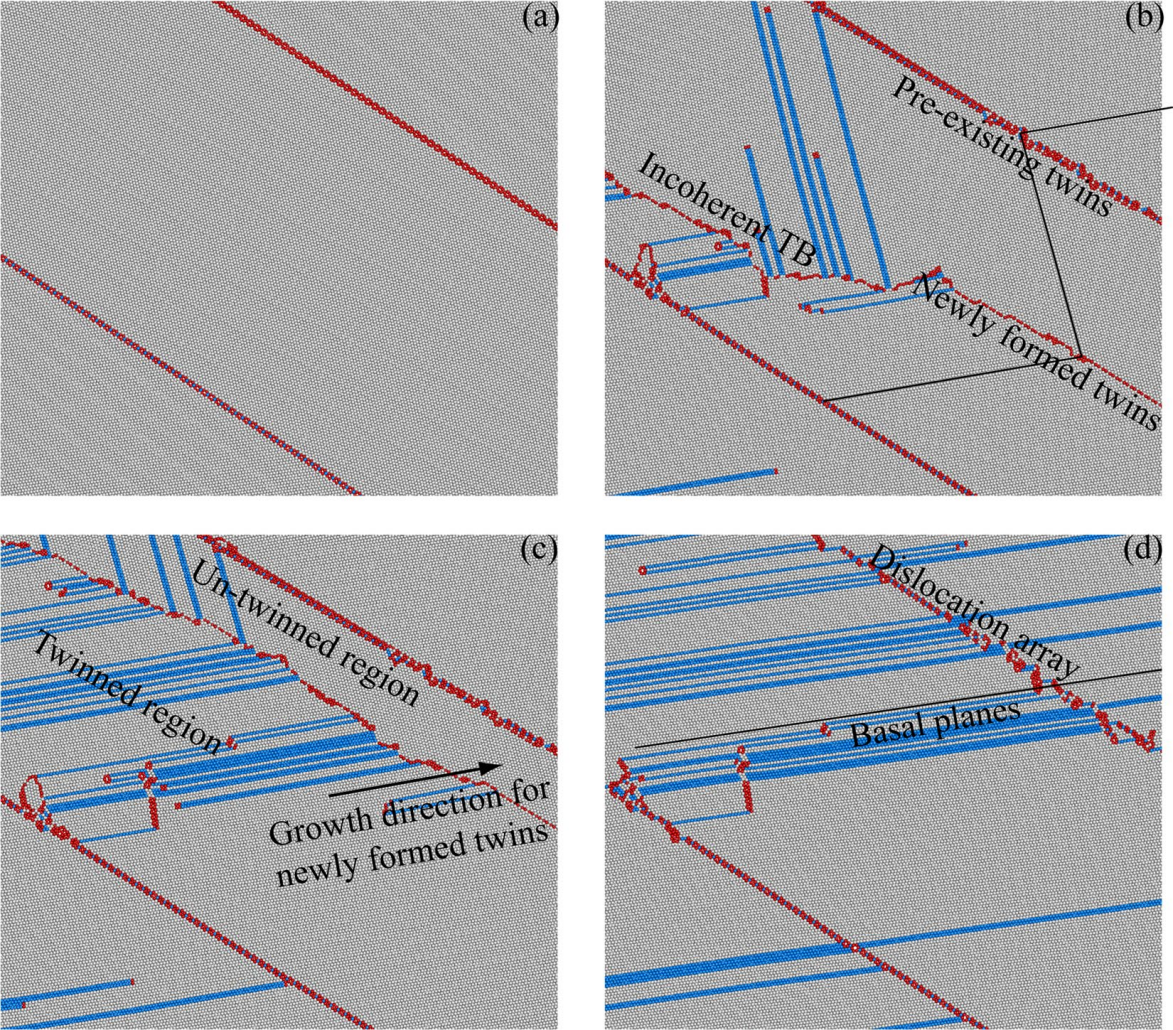
The nucleation and growth of newly formed tensile twins inside one half of the original lamellar tensile twins. The deformation patterns are collected at strains of (**a**) 0%; (**b**) 3%; (**c**) 4%; (**d**) 7%.

**Figure 4:**
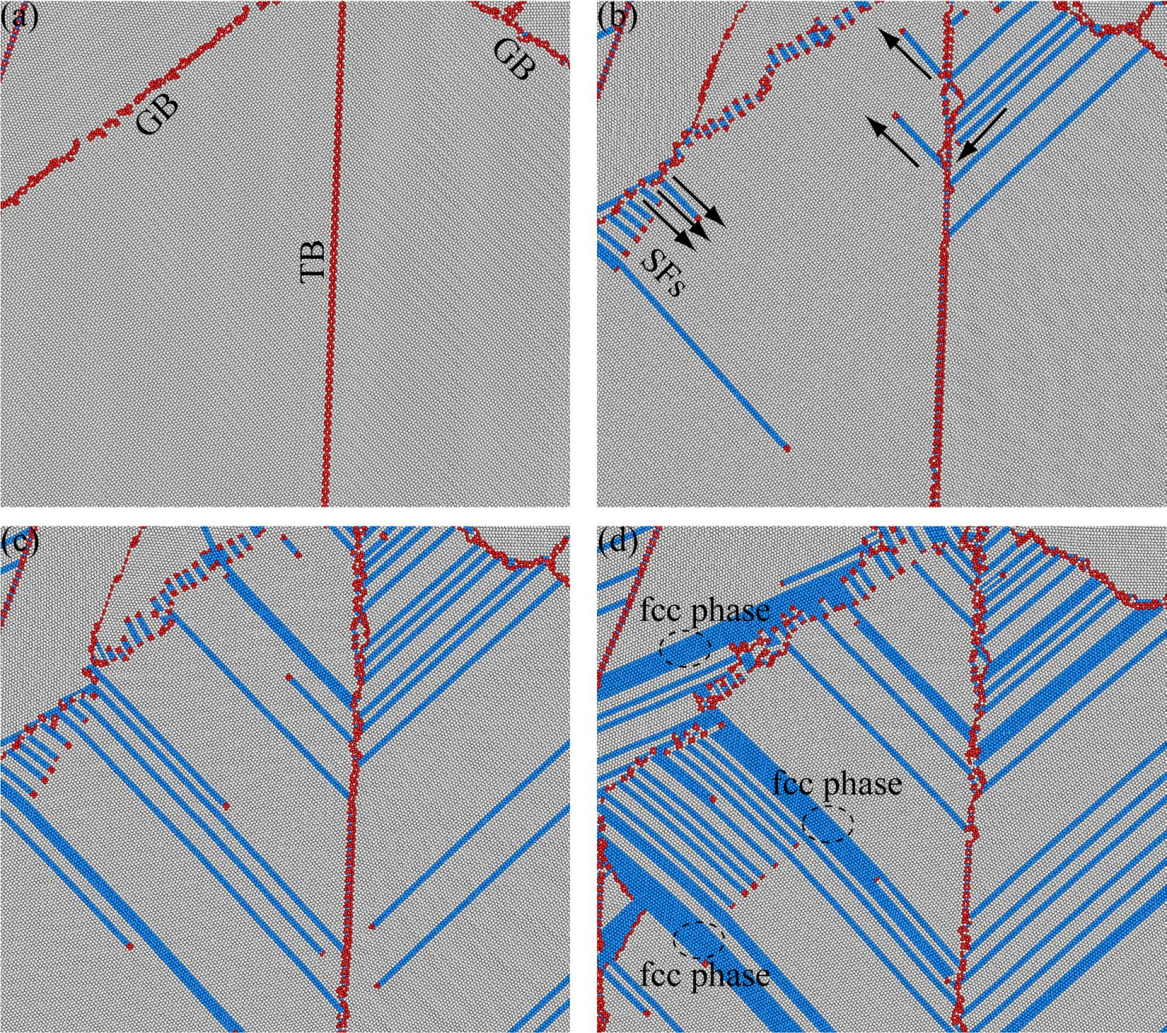
Partial basal dislocations nucleated from GBs/TBs are blocked by other TBs/GBs and phase transformation. The deformation patterns are collected at strains of (**a**) 0%; (**b**) 3%; (**c**) 4%; (**d**) 7%.

**Figure 5:**
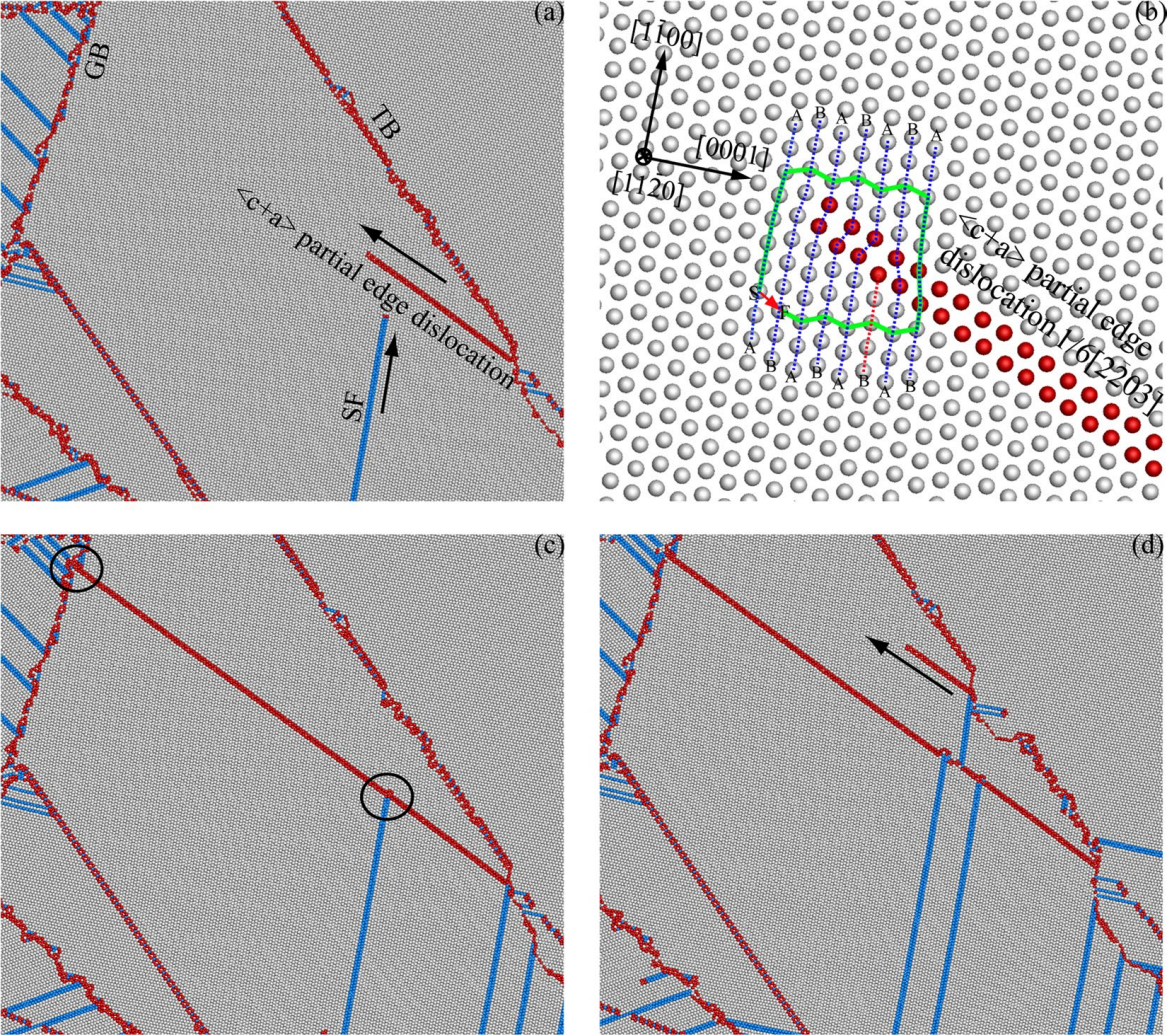
Interaction between <c + a> partial edge dislocation nucleated from TBs and basal partial dislocations. The deformation patterns are collected at strains of (**a**) 3%; (**c**) 4%; (**d**) 5%. (**b**) The Burgers circuit and the Burgers vector for the <c + a> partial edge dislocation 1/6[$2\bar{2}03$].

In MD simulations, snapshots of microstructure evolution at various strains can be easily obtained. In this prospective, the deformed atomistic configurations are provided in order to understand the TBS effect on the flow behaviors of NC hcp cobalt. For NC hcp cobalt either with lamellar {$10\bar{1}2$} tensile twins or lamellar {$10\bar{1}1$} compressive twins, the simulated deformation patterns for both large TBS (TBS = 23.76 nm or 24.59 nm) and small TBS (TBS = 2.38 nm or 2.31 nm) are provided and compared. As shown in Fig. 2, when TBS of the lamellar {$10\bar{1}2$} tensile twins is large (TBS = 23.76 nm), the deformation mechanisms can be categorized into four types: (i) The interactions between partial basal dislocations emitted from grain boundaries (GBs)/TBs and other TBs/GBs; (ii) Phase transformation from hcp to fcc by basal stacking faults (SFs) at adjacent planes; (iii) The interactions between <c + a> partial edge dislocations and basal partial dislocations nucleated from TBs/GBs; (iv) The nucleation and growth of newly formed tensile twins inside one half of the lamellar tensile twins. The details of these deformation mechanisms will be shown in Figs 3–5 by close-up views.

The corresponding close-up views for Fig. 2 showing the nucleation and growth of newly formed tensile twins inside one half of the lamellar tensile twins are displayed in Fig. 3. The newly formed tensile twins are deformation twins by tensile deformation, which are nucleated at GBs and formed by separation and propagation of multiple twinning dislocations from GBs^36^. The previous paper also indicated that the deformation tensile {$10\bar{1}2$} twins could be formed by simultaneous glide of a zonal dislocation consisting of partial dislocation and multiple twinning dislocations^20^. As observed, the newly twinned region can sustain the basal slip more easily and more basal SFs are observed compared to the un-twinned region due to a change of crystallographic orientation from a lower Schmid factor to a higher Schmid factor during the deformation twinning, which should contribute to the strengthening. It should also be interesting to note that the TB for the newly deformation twinning is incoherent. The TB deviates from the {$10\bar{1}2$} twinning plane although the misorientation angle for the newly deformation twinning is close to the theoretical value of 85.8°^36, 40, 41^. These non-classical twinning behaviors could be due to a homogeneous shear plus atomic shuffling, and have also been observed experimentally in dynamically deformed cobalt^41^. These experimental observations, combined with the current results, indicate that the invariant plane strain condition required for the hcp twinning theories breaks down for the tensile deformation twins in cobalt during the plastic deformation. When the newly formed twinned region propagates to the next original TB (Fig. 3d), the two parts of the original tensile twins reconcile into one part with almost zero degree of misorientation angle, left with dislocation arrays at the location of the original TB.

The corresponding close-up views for Fig. 2 showing two following deformation mechanisms are displayed in Fig. 4: (i) Partial basal dislocations nucleated from GBs/TBs interacting with other TBs/GBs and formation of basal SFs; (ii) Phase transformation from hcp phase to fcc phase by basal SFs at adjacent planes. SFs can be formed when partial dislocations are nucleated and slip along the close-packed planes in crystalline metals. SFs generally have an equilibrium width, determined by the balance between the SFE and the repulsive force of the partial dislocations, when a trailing partial dislocation is also nucleated behind SFs. Unlike SFs in fcc metals, SFs in hcp metals are much more complex due to the low symmetry and three slip systems associated with SFs in hcp metals have been confirmed so far by MD simulations^40^. Among them, SFs on basal planes are most likely to be observed due to the relative low SFE for the basal planes. Previous research have indicated that three kinds of basal SFs may occur in hcp metals: two intrinsic SFs (*I*_1_ and *I*_2_) and one extrinsic SF (*E*)^31, 36^. In Fig. 4, the leading basal partial dislocations are found to have an Burgers vector of $2/3[1\bar{1}00]$ and the corresponding SFs are intrinsic (*I*_2_ type)^36^. The density of basal SFs increases with increasing strain, and these deformation-induced basal SFs span through the entire grain with leading/trailing partial dislocations residing in GBs/TBs. As observed, the propagating basal partial dislocations are blocked by TBs/GBs for strain hardening and strengthening, pretty much similar to the corresponding mechanism in fcc metals^42^. Cobalt is a special pure metal, displaying a transformation between fcc and hcp phases with temperature change or plastic deformation^31^. Phase transformation from hcp phase to fcc phase are observed by basal SFs at adjacent planes during the tensile plastic deformation, as shown in Fig. 4d. It is well known that the interfaces between difference phases can lead to a high strength and enhanced strain hardening in nanoscale metallic multilayer systems^43^, thus the formed phase boundaries for fcc/hcp interfaces after phase transformation may provide strengthening due to the possible interactions between glide dislocations and interfaces.

The corresponding close-up views for Fig. 2 showing the nucleation of <c + a> partial edge dislocations from TBs and their interactions with basal partial dislocations are displayed in Fig. 5. The <c + a> dislocations have received much attention and are significant during the homogeneous/uniform plastic deformation in hcp metals because they have been considered as the key factor for the enhanced strain hardening and the improved ductility^22–25, 31, 36^. It is energetically costly for the nucleation of <c + a> slip due to its large Burgers vector, thus higher critical resolved shear stress is required and <c + a> dislocation may occur at positions with high local stresses. As shown in Fig. 5a, an non-basal dislocation is nucleated from the TB and its Burgers circuit is displayed in Fig. 5b. The non-basal dislocation is identified as <c + a> partial edge dislocation with Burgers vector of 1/6[$2\bar{2}03$]. An extra half plane of atoms is produced (as labeled layer B with red dash line in Fig. 5b) by the <c + a> partial edge dislocation. Since this <c + a> dislocation is edge type, its dislocation line is parallel to the *z* axis of $[11\bar{2}0]$, thus a non-basal SF is generated during the propagation of the <c + a> partial edge dislocation. As shown in Fig. 5d, strong interaction between the basal SF and the non-basal SF is observed, and the propagation of the basal SF is blocked by the non-basal SF, resulting in strain hardening behaviors^44^. The propagation of the <c + a> partial edge dislocation is also observed to be blocked by GBs, resulting in additional strain hardening.

When TBS of the lamellar {$10\bar{1}2$} tensile twins is small (TBS = 2.38 nm), the deformation mechanisms are identical and consistent compared to those for large TBS, and the following four deformation mechanisms are also observed (Fig. S2): (i) Partial basal dislocation activities; (ii) Phase transformation; (iii) <c + a> partial edge dislocation activities; (iv) Newly formed tensile twins. As discussed earlier, those four deformation mechanisms should contribute to the strengthening when TBS decreases.

### Atomistic deformation mechanisms for NC hcp cobalt with compressive twins

The deformation patterns for the NC hcp cobalt with lamellar {$10\bar{1}1$} compressive twins (TBS = 24.59 nm) are shown in Fig. S3. When TBS of lamellar {$10\bar{1}1$} compressive twins is large, the deformation mechanisms can also be categorized into four types: (i) Partial basal dislocation activities; (ii) Phase transformation; (iii) <c + a> partial edge dislocation activities; (iv) Formation of secondary tensile twins inside primary compressive twins. The details of the first three deformation mechanisms are similar to those in the NC hcp cobalt with lamellar {$10\bar{1}2$} tensile twins, and are shown in Figs S4, S5 by close-up views.

**Figure 6:**
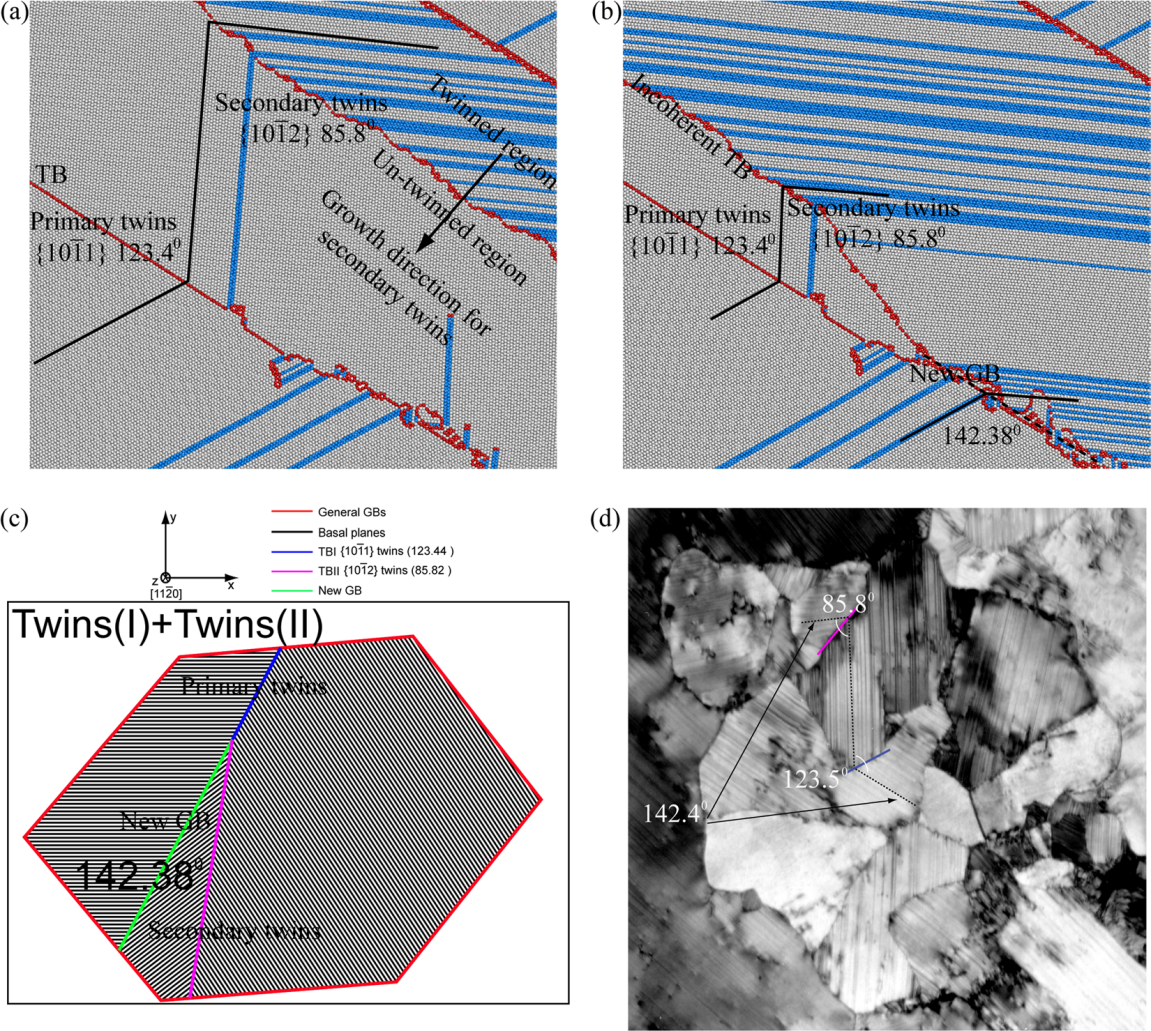
Formation of the secondary tensile twins inside the primary compressive twins. The deformation patterns are collected at strains of (**a**) 3%; (**b**) 4%. (**c**) Schematic of formation sequences of the secondary tensile twins inside the primary compressive twins. (**d**) Experimental TEM observation for the secondary tensile twins generated inside the primary compressive twins.

The corresponding close-up views for Fig. S3 showing the nucleation and growth of secondary tensile twins inside original primary compressive twins are displayed in Fig. 6. Again, the twinned region for the newly formed secondary tensile twins is observed to sustain the basal slip more easily compared to the un-twinned region, and the TB for the newly formed secondary tensile twins is incoherent. The formation of such multiple twinning from the virgin grain is schematically illustrated in Fig. 6c. First, the primary compressive twins could be preset (like in our simulations) or could be formed inside the virgin grain when the material is subjected to compressive deformation, such as rolling, shot peening, and a compressive TB could be formed (named as TBI with blue marked line). This compressive twinning process changes the orientation of the right part of TBI with a angle of 123.44° to the left matrix. Further tensile deformation leads to the formation of the secondary tensile twins (with TBII, purple marked line) inside the right part of the primary compressive twins, thus the lower part should be re-oriented again due to the secondary tensile twinning. Finally, the green marked boundary is newly formed during multiple twinning and becomes the special GB with a misorientation angle of 142.38° at both sides. Previous research also mentioned the all possible angles for the newly formed special GB produced by two or three twinning events in Mg alloy^21^, the angle of the special GB for the two twinning (TBI + TBII) is very similar to the present case.

The multiple twinning processes have also been observed by experiments in NC hcp cobalt. The NC hcp cobalt tensile specimens have been obtained from the surfaces of the samples processed by surface mechanical attrition treatment (SMAT). After the quasic-static tensile testing, the samples were examined by transmission electron microscope. The other experimental details can be found in the Methods section. Several multiple twinning modes (TBI + TBI; TBI + TBI + TBI; TBI + TBII) were identified in the tensile tested samples. The last multiple twinning mode (TBI + TBII) is shown in Fig. 6d, which is similar to the observation from MD simulations (Fig. 6b). As we know, TBI is a compressive twinning mode since it typically forms under compressive stress, while TBII is a tensile twinning mode since it typically forms under tensile loading. SMAT is compressive loading with severe plastic deformation, while the subsequent tensile tests provide tensile stress state. Thus, the first two multiple twinning modes (TBI + TBI; TBI + TBI + TBI) most likely were formed during the SMAT process, while the last multiple twinning mode (TBI + TBII) should be generated during the subsequent tensile testing. When TBS of the lamellar {$10\bar{1}1$} compressive twins is small (TBS = 2.31 nm), the deformation mechanisms are identical and consistent compared to those for large TBS, and the aforementioned four deformation mechanisms (Figs S4–S6) are also observed (Fig. S6). As discussed earlier, those four deformation mechanisms should also contribute to the strengthening when TBS decreases.

## Discussions

As mentioned earlier^14, 39^, the strength of NT fcc metals first increases with decreasing TBS, achieving a maximum value at the critical TBS, and then drops when the TBS further decreases. This is due to a transition in deformation mechanism at the critical TBS for fcc metals^14, 39^, from the interactions between dislocations and TBs with an inclined angle at large TBS to the detwinning with dislocations nucleated parallel to TBs at small TBS. However, there is no transition for deformation mechanisms in NC hcp cobalt with nanotwins when the TBS decreases, thus the strength monotonically increases with decreasing TBS due to the consistent strengthening deformation mechanisms no matter TBS is large or small for lamellar {$10\bar{1}2$} tensile twins or {$10\bar{1}1$} compressive twins.

Lamellar {$10\bar{1}2$} tensile twins or {$10\bar{1}1$} compressive twins could be generated by deformation twins using severe plastic deformation (SPD) or by growth twins using electroplated process. These pre-existing twins will play very important roles in the following tensile deformation, contributing to the strengthening all way to very small TBS and to strain hardening by interactions between dislocations and TBs and phase transformation. More importantly, multiple twinning could be formed during subsequent tensile deformation, which plays a key role for strengthening and strain hardening in the plastic deformation for hcp metals with low SFE^21, 32^. Overall, four different deformation mechanisms are observed in the NC cobalt with lamellar compressive/tensile twins, and these consistent mechanisms with decreasing TBS will contribute to the strengthening and the strain hardening in such a way as discussed in following: (i) Additional TBs for pre-existing lamellar twins should pose extra energy barriers for the basal partial dislocations to overcome, which is very similar to the TB strengthening in fcc metals. High density of dislocations thus are stored between preexisting TBs, resulting in also dislocation accumulation for strain hardening. (ii) Phase transformation will create numerous phase boundaries, and these phase interfaces should also pose strong energy barriers for gliding dislocations in other slip systems to overcome. The blocking of dislocations by phase interfaces will on one hand hinder the slip of dislocations for strengthening, and on the other hand will accumulate dislocations between interfaces for strain hardening (iii) Lots of <c + a> partial edge dislocations are observed to nucleate, and these non-basal dislocations not only impede the motion of the basal partial dislocations for strengthening, but also strongly interact with preexisting TBs/GBs for strain hardening. (iv) Formation of secondary twins inside preexisting primary twins should change the crystallographic orientation from a lower Schmid factor to a higher Schmid factor for the corresponding region, thus promoting more dislocations in this region for strain hardening. So, lamellar {$10\bar{1}2$} tensile twins or {$10\bar{1}1$} compressive twins with TBS to very small values could be generated in NC hcp metals with low SFE to achieve both high strength by aforementioned strengthening mechanisms and high ductility by enhancing strain hardening rate through the four mechanisms mentioned above. While aforementioned four deformation mechanisms all operate in the NC hcp metals with lamellar {$10\bar{1}2$} tensile twins or {$10\bar{1}1$} compressive twins, their contributions to the overall strength should be different and may vary with the size of the lamellar twins and the grain size. Although it is difficult to quantify their contributions, more studies should be conducted in future work to identify the major source of strengthening by varying both grain size and lamellar twin thickness.

In summary, a series of large-scale MD simulations have been performed to investigate the TBS effects of {$10\bar{1}2$} tensile/{$10\bar{1}1$} compressive twins on the tensile strength and the corresponding atomistic deformation mechanisms of NC hcp cobalt, the main finding are summarized as follows. The strength monotonically increases with decreasing TBS and no softening stage is observed for NC hcp cobalt with both lamellar {$10\bar{1}2$} tensile twins and lamellar {$10\bar{1}1$} compressive twins, totally different from that for fcc metals. No transition for deformation mechanisms is found in NC hcp cobalt when the TBS decreases, thus the TBS effect on the strength is due to the consistent four types of deformation mechanisms. One of four types of deformation mechanisms, multiple twinning, has also been observed by TEM after tensile testing in NC hcp cobalt obtained by severe plastic deformation, and the formation mechanisms and sequences are found to be consistent with MD simulations. The observed four deformation mechanisms by nanoscale twins should contribute to the strengthening and the strain hardening when TBS decreases. The finding in the present results should acquire a better understanding for the strengthening of lamellar twins and provide insights to design the microstructures for reinforcing the mechanical properties in the hcp metals with low SFE.

## Methods

### Procedures for MD simulations

The MD simulations have been performed by the Large-scale Atomic/Molecular Massively Parallel Simulator (LAMMPS) and a Co EAM potential developed by Pun and Mishin^45^. This potential is calibrated according to the experimental results and the *ab initio* calculations for many basic properties, including the lattice constants, elastic constants, stacking faults energies, vacancy formation and migration energies, surface energies, cohesive energies. In order to explore the plastic deformation mechanisms of nanocrystalline cobalt with lamellar twins, it is necessary to consider simulation cells with grains larger than those possible in fully 3-dimensional simulations. In this perspective, $[11\bar{2}0]$-textured simulation cells with hexagonal columnar grains (Similar to the configuration used by Kim *et al*.^36^) were considered in the present study. The crystallographic orientation of the columnar axis was carefully selected to be $[11\bar{2}0]$ in order to allow various dislocation processes^36^, such as basal slip, non-basal slip (including <*c* + *a*> dislo*ca*tions) and various deformation twins. The thickness of the simulation cells is 5.0*a*_0_ (*a*_0_ = 0.2519 nm), cont*a*ining 10 atomic planes (*z* direction). The grains are separated from each other by high-angle tilt GBs with rotation angles about the columnar axis. The dimensions of the simulation cells are 240 × 240 ×1.26 nm^3^, which contain approximately 7,160,000 atoms. Lamellar {$10\bar{1}2$} tensile twins and lamelalr {$10\bar{1}1$} compressive twins are generated inside nano-grains by mirror symmetry about the {$10\bar{1}2$} plane and the {$10\bar{1}1$} plane, respectively. It should be noted that the two half parts of the tensile twins and the compressive twins satisfy 85.8° <$10\bar{1}2$> and 123.4° <$10\bar{1}1$> orientation relationship, respectively. The atoms are colored based on common neighbor analysis (CNA) values in the present study. Gray color stands for perfect hcp atoms, blue color stands for fcc atoms and red color is for other atoms which belong to grain boundaries (GBs), free surfaces or other defects. Periodic boundary conditions were imposed for all directions. Before tensile loading, the as-constructed simulation cells were first subjected to energy minimization by the conjugate gradient method, and then heated up to the desired temperature (10 K) and finally relaxed by the Nose/Hoover isobaric-isothermal ensemble (NPT) under both the pressure 0 bar and the temperature 10 K for 100 ps. After relaxation, the simulation cell was stretched along *x*-axis with a constant strain rate of 5 × 10^8^ *s* ^−1^ for a strain of 10%. The uniaxial loading condition was kept during the tensile loading by setting the pressures in the *y* and *z* directions to be zero.

### NC cobalt preparation, tensile tests and TEM observations

In the present paper, CG cobalt plates with high purity (wt% 99.99) were first produced by electro-deposition, and have a thickness of 5 mm and an average grain size of ~30 μm with pure hcp crystal structure. Then a microstructure with grain size gradient in cobalt was produced by the surface mechanical attrition treatment (SMAT) technique. The details of this technique have been described elsewhere^31^. Dogbone-shaped tensile specimens with NG structure (thin specimens with a thickness of 50 μm from the top surfaces of SMATed samples) were prepared by mechanically ground and electro-polished to a mirror finish. The dimensions of the gauge sectional area for the designed tensile samples are 8 mm long × 2.5 mm wide. Tensile tests were performed at a strain rate of 4 × 10^−4^ s^−1^, and the samples were examined in a transmission electron microscope (TEM) after tensile testing. TEM specimens for *ex-situ* observations were cut from the gauge section of the tensile sample, and prepared by conventional twin-jet polishing technique.

## Electronic supplementary material

Supporting information
