# Supplementary material for: Size effects of lamellar twins on the strength and deformation mechanisms of nanocrystalline hcp cobalt
Source: Sci Rep. 2017 Aug 25;7:9550. doi: 10.1038/s41598-017-09919-2 (PMC5573393; doi:10.1038/s41598-017-09919-2)
Supplement: Supplementary file 1 — Supporting information [file 41598_2017_9919_MOESM1_ESM.doc]

**Size effects of lamellar twins on the strength and deformation mechanisms of nanocrystalline hcp cobalt**

Wen Wang^1,2^, Fuping Yuan^1,2*^, Ping Jiang^1^ & Xiaolei Wu^1,2^

^1^ State Key Laboratory of Nonlinear Mechanics, Institute of Mechanics, Chinese Academy of Sciences, Beijing 100190, China

^2^School of Engineering Science, University of Chinese Academy of Sciences, Beijing 100190, People’s Republic of China

**Supplementary Information**


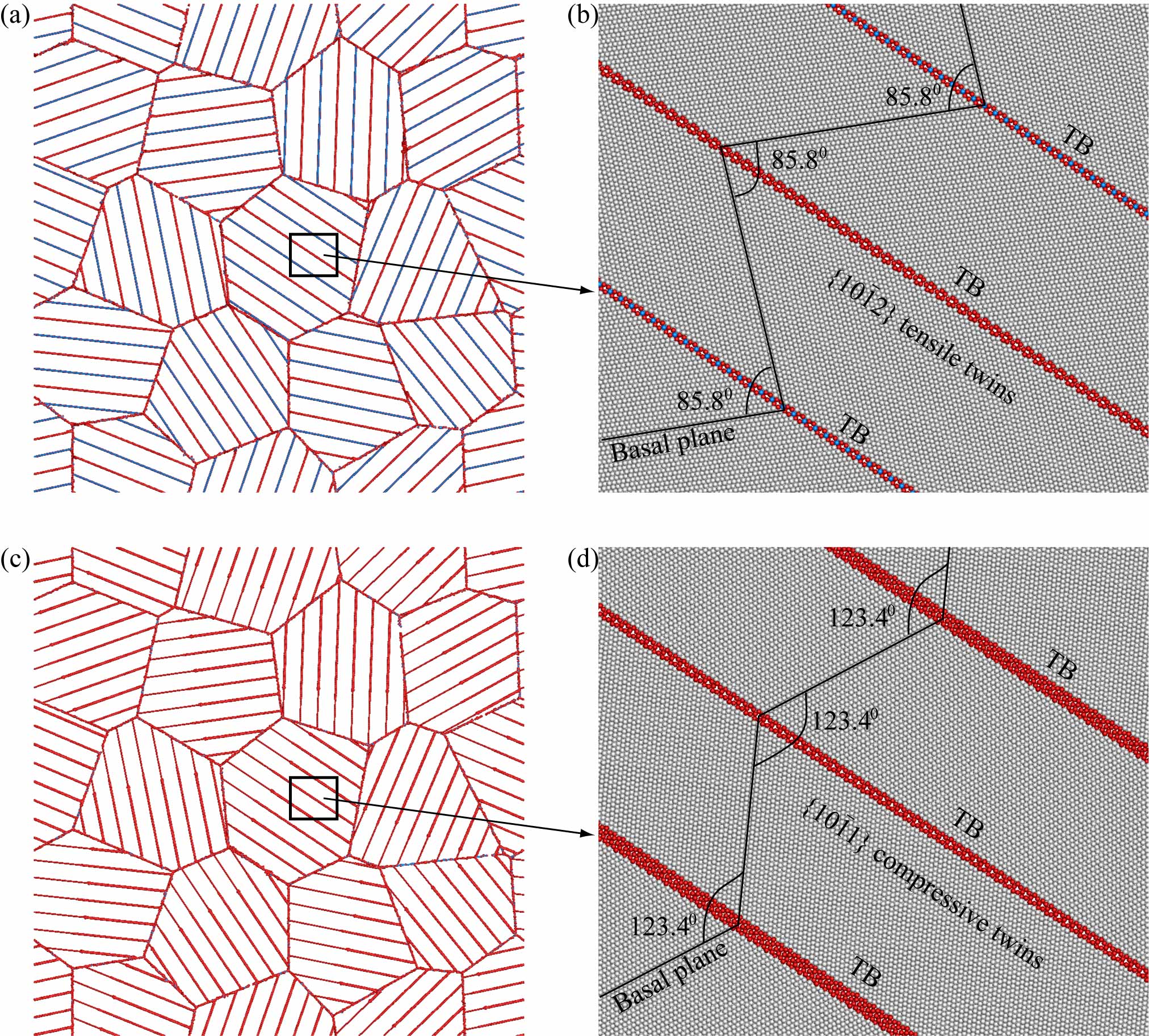


Figure S1. The $[11\bar{2}0]$-textured simulation cells with hexagonal columnar grains and lamellar twins. (a) (c) The configurations for NC hcp cobalt (*d* = 60 nm) with lamellar {$10\bar{1}2$} tensile twins (TBS = 8.32 nm) and with lamellar {$10\bar{1}1$} compressive twins (TBS = 8.45 nm), respectively; (b) (d) The corresponding close-up views for the rectangular areas in Figs. S1a and S1c showing the details for the lamellar {$10\bar{1}2$} tensile twins and the lamellar {$10\bar{1}1$} compressive twins, respectively.


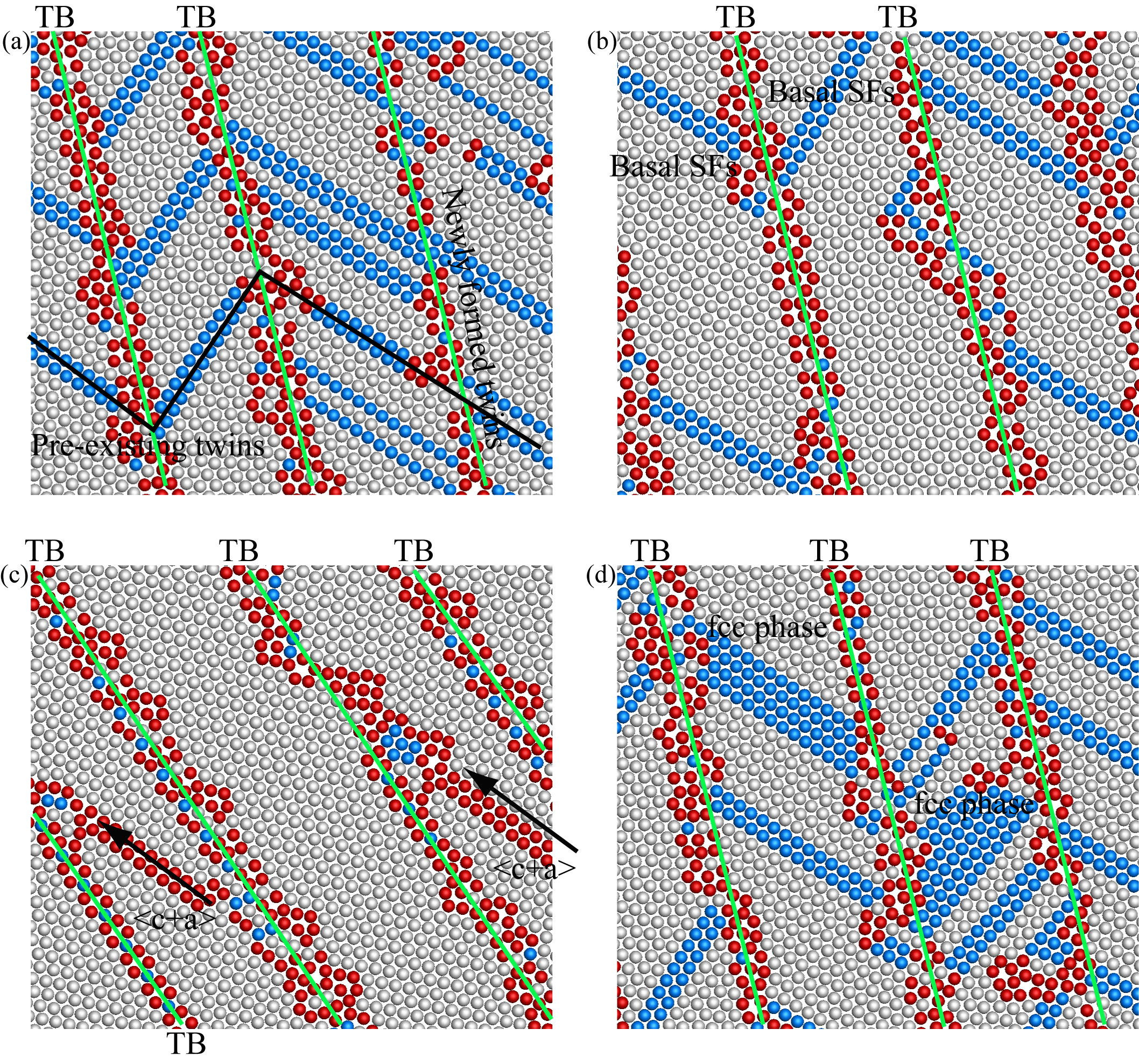


Figure S2. Simulated deformation pattern for nanocrystalline cobalt with lamellar {$10\bar{1}2$} tensile twins (TBS = 2.38 nm) at strain of 5% showing the various deformation mechanisms: (a) Newly formed tensile twins; (b) Partial basal dislocation activities; (c) <c+a> partial edge dislocation activities; (d) phase transformation.


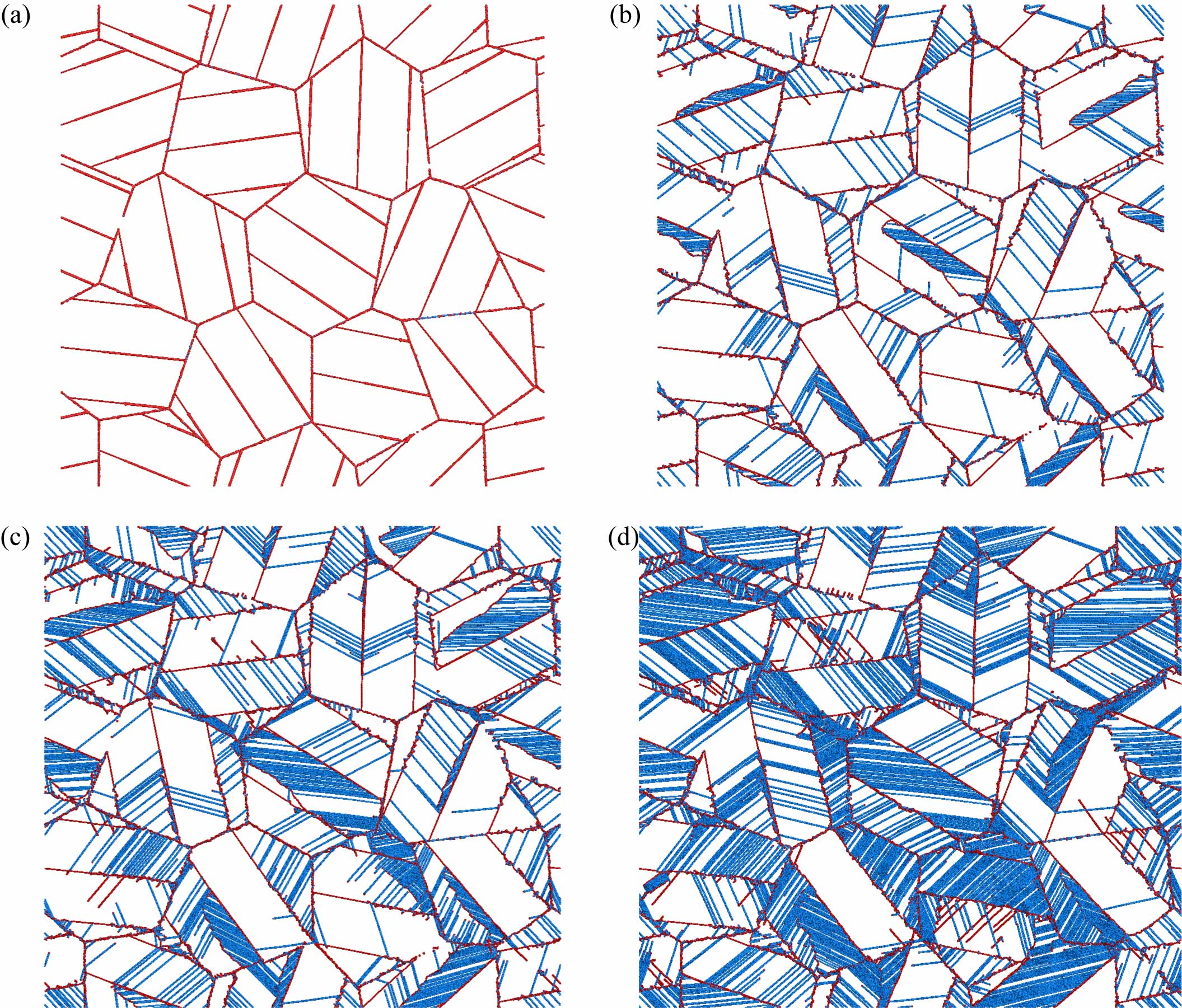


Figure S3. Simulated deformation patterns for nanocrystalline cobalt with lamellar {$10\bar{1}1$} compressive twins (TBS = 24.59 nm) at strains of (a) 0%; (b) 3%; (c) 4%; (d) 7%.


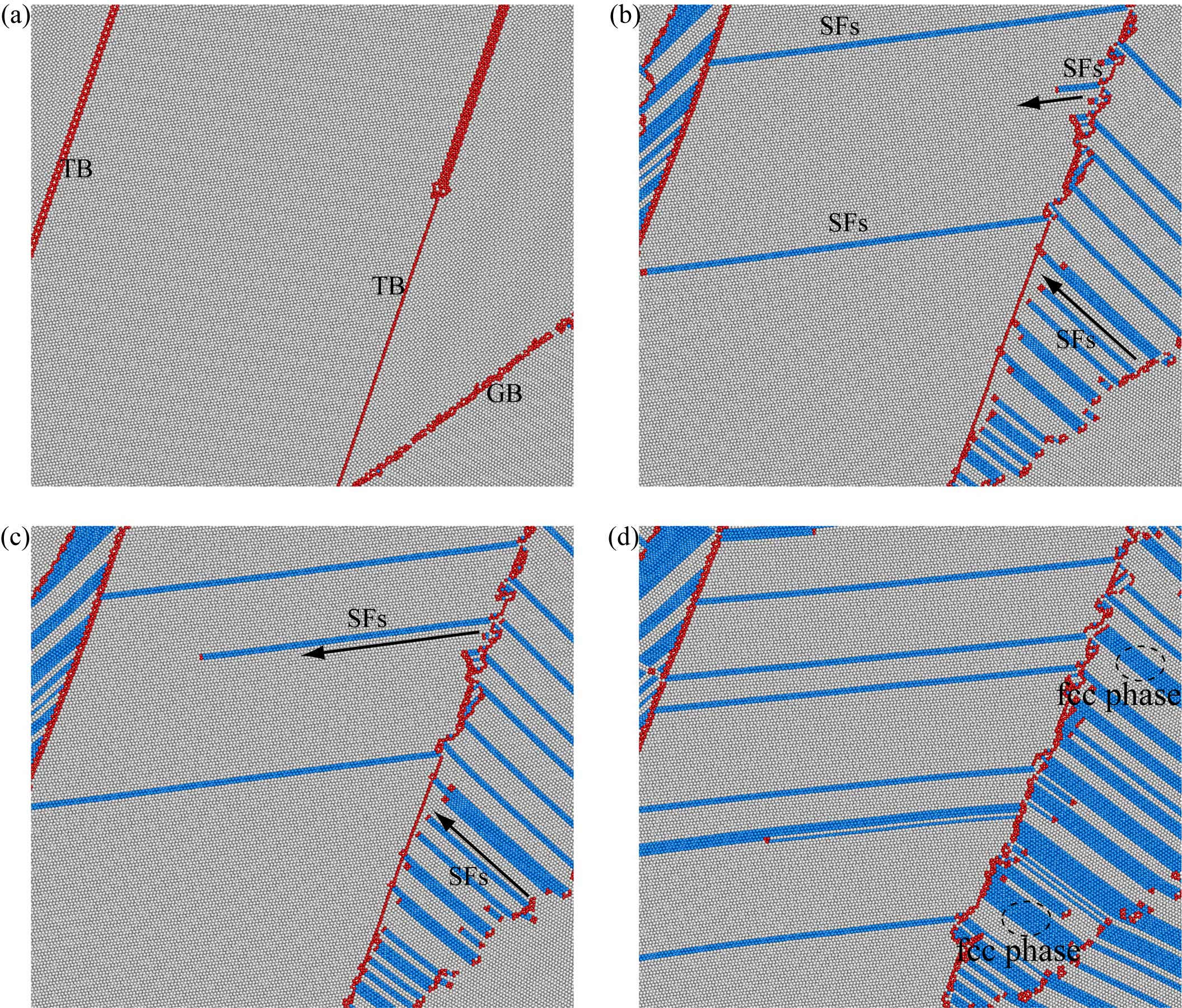


Figure S4. The corresponding close-up views for Fig. S3 showing the following two deformation mechanisms: Partial basal dislocations nucleated from GBs/TBs are blocked by TBs/GBs; Phase transformation. The deformation patterns are collected at strains of (a) 0%; (b) 3%; (c) 4%; (d) 7%.


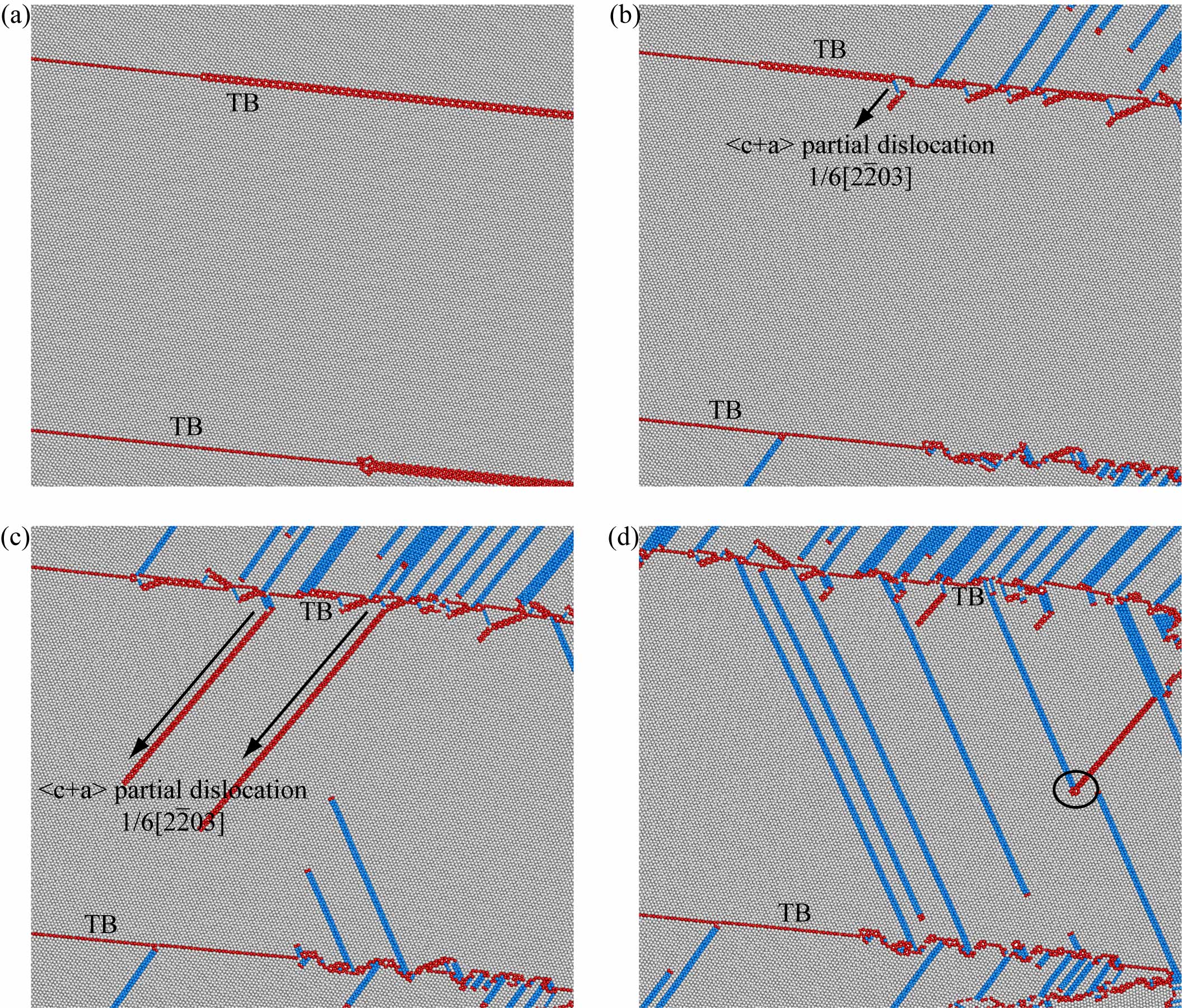


Figure S5. The corresponding close-up views for Fig. S3 showing the following deformation mechanism: Interaction between <c+a> partial edge dislocations nucleated from TBs and basal partial dislocations. The deformation patterns are collected at strains of (a) 0%; (b) 3%; (c) 4%; (d) 7%.


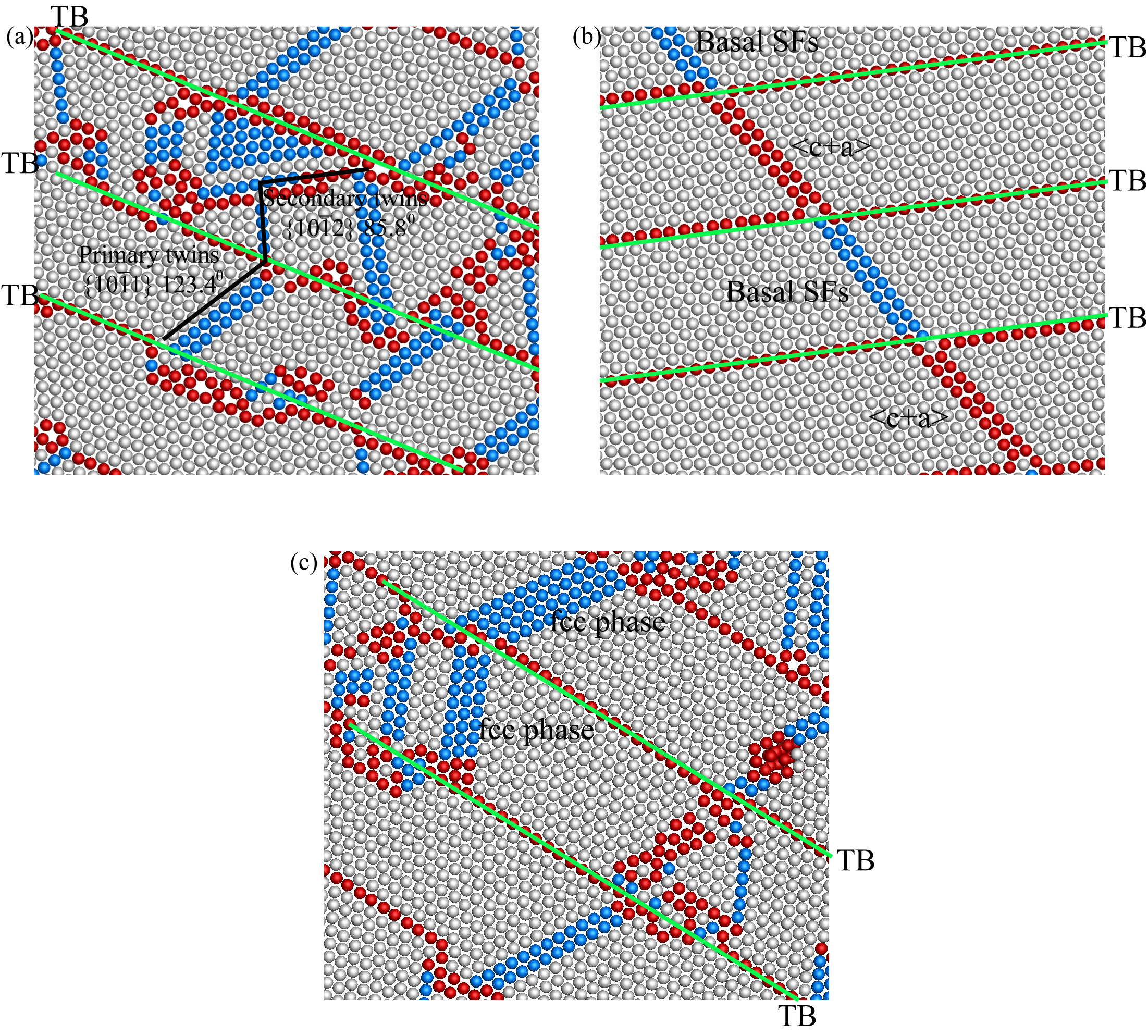


Figure S6. Simulated deformation patterns for nanocrystalline cobalt with lamellar {$10\bar{1}1$} compressive twins (TBS = 2.31 nm) at strain of 4% showing the various deformation mechanisms: (a) Formation of the secondary tensile twins inside the primary compressive twins; (b) Partial basal dislocation activities and <c+a> partial edge dislocation activities; (c) Phase transformation.
